# Clinical Impact of ACE-I/ARB for Conservatively Treated Patients with Moderate to Severe Mitral Regurgitation: A Single Center Observational Study

**DOI:** 10.3390/jcdd10040177

**Published:** 2023-04-18

**Authors:** Robert Uzel, Raphael R. Bruno, Christian Jung, Christian Lang, Hannes Hoi, Martin Grünbart, Christian Datz, Friedrich Hoppichler, Bernhard Wernly

**Affiliations:** 1 Department of Internal Medicine, Saint John of God Hospital, Teaching Hospital of the Paracelsus Medical Private University, Kajetanerplatz 1, 5020 Salzburg, Austria; 2 Department of Cardiology, Klinik Floridsdorf, Brünner Straße 68, 1210 Vienna, Austria; 3 Department of Cardiology, Pulmonology and Vascular Medicine, Medical Faculty, Heinrich-Heine-University Duesseldorf, Moorenstraße 5, 40225 Duesseldorf, Germany; 4 Department of Pulmonology, Medical University of Vienna, Waehringer Guertel 18-20, 1090 Vienna, Austria; 5 Department of Surgery, Saint John of God Hospital, Teaching Hospital of the Paracelsus Medical Private University, Kajetanerplatz 1, 5020 Salzburg, Austria; 6 Department of Internal Medicine, General Hospital Oberndorf, Teaching Hospital of the Paracelsus Medical Private University, Paracelsusstraße 37, 5110 Oberndorf, Austria; 7 Special Institute for Preventive Cardiology and Nutrition, SIPCAN—Initiative für ein gesundes Leben, 5020 Salzburg, Austria; 8 Institute of General Practice, Family Medicine and Preventive Medicine, Paracelsus Medical University, Strubergasse 21, 5020 Salzburg, Austria

**Keywords:** mitral regurgitation, HFmrEF, ACE inhibitor, angiotensin receptor blocker, heart failure

## Abstract

(1) Background: Mitral regurgitation (MR) is associated with increased mortality and frequent hospital admissions. Although mitral valve intervention offers improved clinical outcomes for MR, it is not feasible in many cases. Moreover, conservative therapeutic opportunities remain limited. The aim of this study was to evaluate the impact of ACE inhibitors and angiotensin receptor blockers (ACE-I/ARB) on elderly patients with moderate-to-severe MR and mildly reduced to preserved ejection fraction. (2) Methods: In total, 176 patients were included in our hypothesis-generating, single-center observational study. Hospitalization for heart failure and all-cause death have been defined as the combined 1-year primary endpoint. (3) Results: Patients treated with ACE-I/ARB showed a lower risk for the combined endpoint of death and heart failure-related readmission (HR 0.52 95%CI 0.27–0.99; *p* = 0.046), even after adjustment for EUROScoreII and frailty (HR 0.52 95%CI 0.27–0.99; *p* = 0.049) (4) Conclusions: The use of an ACE-I/ARB in patients with moderate-to-severe MR and preserved to mildly reduced left-ventricular ejection fraction (LVEF) significantly associates with improved clinical outcome and might be indicated as a valuable therapeutic option in conservatively treated patients.

## 1. Introduction

MR is the second most common valvular heart disease in elderly patients and is associated with increased mortality and frequent heart failure-associated hospital admissions, independent of left-ventricular ejection fraction (LVEF) and comorbidities [1,2]. In general, about 50% of the elderly population is affected by valvular heart diseases [3]. In the case of the nonrheumatic primary MR, which is mostly caused by leaflet abnormalities, such as mitral valve prolapse (MVP), the Global Burden of Cardiovascular Disease study estimates 24.2 million prevalent cases [4,5]. Primary MR represents mostly a mechanical problem, which requires surgical treatment in the case of severe symptomatic MR and acceptable surgical risk [2] as surgery is associated with improved survival [6] For inoperable patients or patients at high surgical risk, the ESC and American Heart Association (AHA) guidelines suggest a transcatheter edge-to-edge repair (TEER) at a recommendation class IIB if echocardiographic parameters are fulfilled [2].

In contrast, secondary MR is mostly caused by left-ventricular or left-atrial pathologies, be it either of ischemic or non-ischemic origin (e.g., dilated cardiomyopathy) [7]. The valve leaflets are structurally normal and the coaptation is secondarily impaired by an imbalance between tethering and closing forces [2]. Although there is no data for the prevalence of secondary MR, it is estimated to cause up to 65% of all cases with moderate to severe MR [8,9]. The therapeutic approach for patients with secondary MR with reduced LVEF includes guideline-directed medical therapy followed by cardiac resynchronization therapy and mitral valve intervention such as surgery or transcatheter approaches in appropriate cases [2,7]. TEER should be considered in patients not appropriate for surgery, who meet the relevant criteria and who fulfil echocardiographic parameters [10]. However, there is no specific conservative therapy for patients with MR and preserved or mildly reduced ejection fraction so far. The focus on these patients comprises the control of risk factors and rhythm in case of atrial fibrillation [11]. Notably, in real-life cohorts, the treatment of both primary and secondary MR is challenging and there is a considerable proportion of up to 49% in patients with appropriate indication, who do not undergo surgery or TEER [12,13]. The most frequent characteristics for denied interventions are reduced LVEF, age, and an increased Charlson comorbidity index [13]. The aim of our study was to evaluate whether patients with moderate-to-severe MR and mildly reduced-to-preserved ejection fraction, who are not eligible for intervention or do not have an indication for intervention yet, benefit from therapy with ACE-I/ARB.

## 2. Materials and Methods

**Figure 1:**
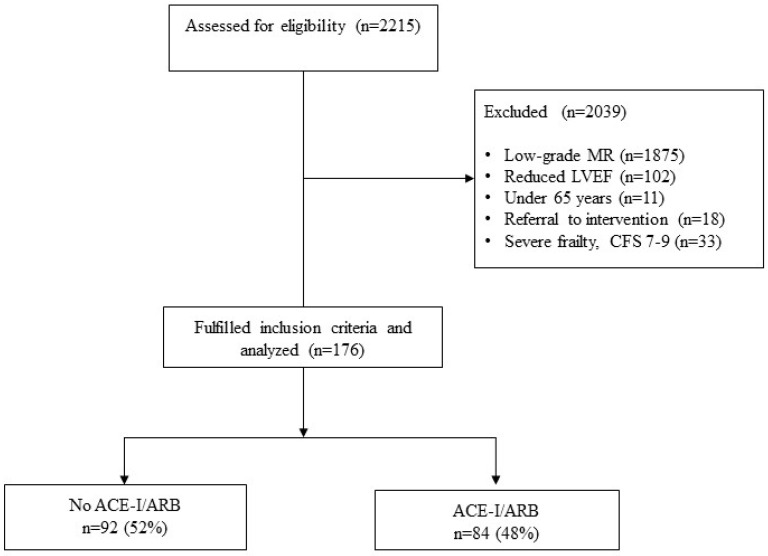
Flowchart of patient inclusion. Abbreviations: MR mitral regurgitation, LVEF left ventricular ejection fraction, CFS clinical frailty scale, ACE-I/ARB ACE-inhibitors and angiotensin receptor blockers.

This retrospective, single-center observational study was approved by the local ethics committee (1170/2020). The study period was selected for 1 January 2014–30 September 2020. The combined primary endpoint was hospitalization due to heart failure and all-cause death. Adults aged 65 years and older, who were admitted to our hospital were queried from the internal database by searching for applicable ICD10 diagnoses related to MR, which resulted in 2215 eligible cases. 2039 were excluded for either low-grade MR, reduced EF, age, or referral to intervention. Since the efficacy of ACE-I/ARB for patients with heart failure and reduced ejection fraction is sufficiently proven, we excluded these patients. The term intervention in this study is used for both surgery and transcatheter approaches. Patients with acute severe mitral regurgitation were excluded since all of them were referred to surgery or TEER. All patients underwent echocardiography in the first days after admission. We included all patients with moderate-to-severe MR, according to European Society of Cardiology guidelines for echocardiography [14]. For the left-ventricular ejection fraction, we used the classification of the European Society of Cardiology [15]. Reduced LVEF is defined as ≤40%, and patients with LVEF ≥50% were considered as “preserved”. Mildly reduced EF is classified as an ejection fraction between 40- and 49%. Further exclusion criteria were in-hospital death, low-grade mitral insufficiency, severe frailty, classified as CFS 7–9, and patients who underwent mitral valve surgery or TEER. A flowchart of patient inclusion is depicted in Figure 1.

We used a comprehensive chart review of our all-digital patient record to obtain pharmacological therapy at discharge, administrative and echocardiographic data. Heart rate at admission and dismissal was evaluated either with a recent 24 h-ECG or the average heart rate in the last 2 days before discharge. Frailty was assessed using the Clinical Frailty Scale, which was gathered from the nursing documentation at hospital admission. The results of the clinical frailty scale were categorized into three groups (well: clinical frailty scale group 1–3, mildly frail: group 4–6, and severely frail: group 7–9). The observation period was shortened to a follow up of one year to reduce the confounding effects of the competing risk. For adjustment, we used EuroScoreII as a multidimensional parameter, which is routinely assessed, has a high degree of familiarity, and includes several relevant comorbidities, in awareness of the fact that it is not validated in this setting.

Baseline characteristics were expressed as means ± standard deviation (SD) for continuous variables, the differences between groups were calculated using U-test. For the expression of categorial variables, we used frequencies, percentages, and the Chi-square test to calculate differences between groups. All-cause death and hospitalization due to heart failure were visualized with Kaplan–Meier plots in Figure 1. We fitted three Cox regression models. The first model-1 was the univariate regression with ACE/ARB as an independent variable, the second model-2 adjusted for both EuroScoreII and frailty, and the third model-3 adjusted for EuroScoreII, frailty as well as aortic stenosis. We obtained hazard ratios (HR) and respective 95% confidence intervals (CI). A HR < 1 suggests a decrease in the risk of hospitalization or death. All tests were two-sided, and a *p*-Value of <0.05 was considered statistically significant. Since not all parameters were available for all categories, patients had to be excluded from the subgroup analyses. For this reason, not all patient numbers add up to 100% (see tables). IBM SPSS Statistics Version 25 and Stata 17 (StataCorp LLC) were used for all statistical computations.

## 3. Results

A total of 176 patients with moderate (70%) or severe (30%) MR were included, of whom 84 (48%) were on treatment with ACE-I/ARB. The baseline characteristics of the study cohort are represented in Table 1. Notably, with the exception of dementia and polypharmacy, clinical demographics did not significantly differ between the control and intervention groups. The most frequent reasons for hospital admission were heart failure, miscellaneous cardiac diseases, or other medical disorders such as infections, malignancies, or gastroenterologic diseases. Groups did not differ significantly in the reason for hospital admission (*p* = 0.69). Patients without ACE-I/ARB suffered more often from dementia (14% vs. 5%; *p* = 0.034). Both groups contained predominantly female patients (66% vs. 65%; *p* = 0.91). There was no significant difference in EuroSCOREII as a multidimensional parameter (7 ± 8 vs. 6 ± 7; *p* = 0.49) nor in individual cardiovascular diseases. Patients with ACE-I/ARB were more often affected by polypharmacy (74% vs. 93%; *p* < 0.001). A total of 41% of patients without ACE-I/ARB had an NYHA level of at least II at baseline, compared to 50% of patients with ACE-I/ARB (*p* = 0.56).

Groups did not differ significantly in baseline echocardiographic parameters as depicted in Table 2. There is a trend toward a higher proportion of aortic stenosis in the group without ACE-I/ARB (26% vs. 17%; *p* = 0.06). Severe aortic stenosis was found in 6%, with a lower proportion in the group with ACE-I/ARB (10% vs. 1%; *p* = 0.01). The majority of patients (70%) suffered from moderate MR.

**Figure 2:**
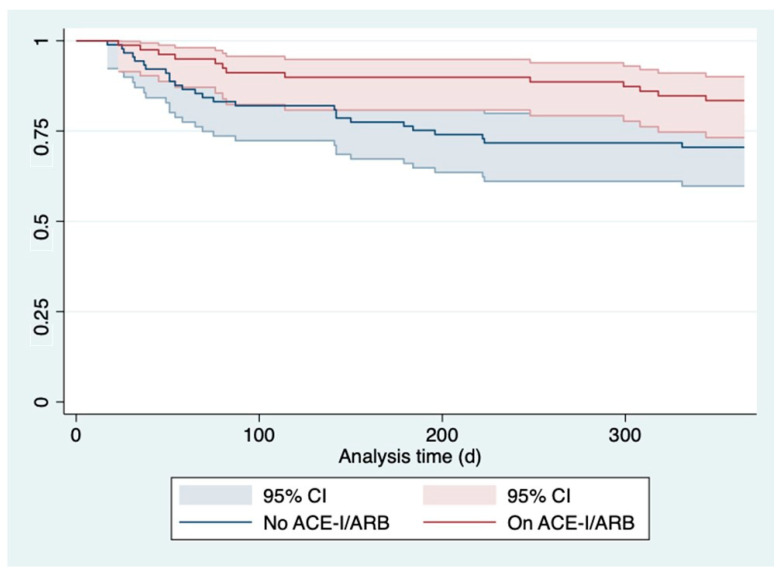
Kaplan–Meier curve illustrating all-cause death or hospitalization due to heart failure dependent on intake of ACE-I/ARB (red line) versus no intake (blue line). Abbreviations: ACE-I ACE-inhibitor; ARB angiotensin receptor blocker.

In both groups, no intervention was performed during the follow up since this was defined as exclusion criteria, as already mentioned in the methods section. The most common arguments for denied intervention in our case were frailty, patients request, an increased Charlson comorbidity index, and age. The Kaplan–Meier curve in Figure 2 depicts all-cause death or hospitalization due to heart failure of patients with versus without ACE-I/ARB and revealed significant differences (*p* = 0.046). Patients on treatment with ACE-I/ARB evidenced a lower risk for the combined endpoint of death and heart failure-related readmission (HR 0.52 95%CI 0.27–0.99; *p* = 0.046) in the univariate model-1.

In model-2, we adjusted for both EuroScoreII and frailty (HR 0.52 95%CI 0.27–0.99; *p*= 0.049), see the multivariable model in Table 3.

Further, we fitted another additional multivariable model-3 adjusting the effect of ACE/ARB on the combined outcome for EuroScoreII, frailty as well as aortic stenosis. We found ACE/ARB to remain independently associated with the combined endpoint (HR 0.51; 95%CI 0.26 to 1.01; *p* = 0.053) at least in trend as depicted in multivariable model 2 in Table 3.

## 4. Discussion

In this hypothesis-generating study, we aimed to evaluate whether the use of ACE-I/ARB is accompanied by an outcome benefit in a real-world collective of patients with moderate-to-severe MR and preserved or mildly reduced LVEF. We excluded patients who were referred to intervention and who suffered from severe frailty (CFS7–9). Patients treated conservatively with ACE-I/ARB evidenced a lower risk for the combined endpoint of death and heart failure-related readmission (HR 0.47 95%CI 0.27–0.99; *p* = 0.047). This effect remained statistically significant after adjustment for EuroScoreII and frailty (HR 0.52 95%CI 0.27–0.99; *p* = 0.049). After further adjustment for EuroScoreII, frailty and aortic stenosis we evidenced a lower risk for the combined endpoint (HR 0.51; 95%CI 0.26 to 1.01; *p* = 0.053), at least in trend.

According to current guidelines, there is still no evidence-based recommendation for pharmacological therapy in patients with chronic MR and mildly reduced or preserved LVEF [2]. The class IIB recommendation for ACE-I/ARB for medical treatment of HFmrEF reflects the current lack of definite evidence. Surgery or TEER are recommended in case of acute severe or symptomatic MR or specific echocardiographic parameters [2]. Nevertheless, according to the reports of the Euro Heart Survey, surgery was not warranted in 49% of patients with severe symptomatic MR [13]. The most striking characteristics were reduced LVEF, age, and comorbidities, since this population faces adverse outcomes [13]. TEER would be a safe alternative in these patients with high surgical risk [16] However, the rate of interventions in mitral regurgitation, when indicated, is low and classified as undertreated [12]. In contrast to a few interventions, there is an overuse of medical therapy in patients with chronic primary MR in absence of guideline recommendations [12,17]. In our sample, only 18 out of 227 patients were referred to surgery or TEER, the majority, old patients with substantial comorbidities, obtained medical treatment. Considering the quantity of the group on conservative treatment and in light of the fact that watchful waiting is a safe strategy in asymptomatic patients, further therapeutic approaches are warranted [2].

The pathomechanism of heart failure in chronic mitral regurgitation is explained by a long-term volume overload on the left ventricle and subsequently its remodeling [18]. Several previous studies investigated the effect of ACE-I/ARBs as these substances cause an afterload reduction accompanied by a decrease in backflow and wall stress [19,20,21]. Most of these studies focused on the change of regurgitation fraction (RF), such as a meta-analysis on randomized controlled trials by Strauss et al., showing an average decrease in RF of 6% [18]. This theory of afterload reduction would be supported by a study by Harris et al., identifying a subgroup of patients with systolic blood pressure ≥ 140 mm, which was less likely to progress and showed a reduction in MR severity under treatment with ACE-I [22]. Volume overload and transforming growth factors are discussed to play a part in MR since they have a possible impact on valve myxomatous degeneration. Malev et al. showed that patients on ARB experienced less degeneration on their mitral valves. However, as in the previous-mentioned studies, there was no effect of ARB on LVEF [23].

Unfortunately, large-sized and randomized clinical trials in this field are missing since the current focus of research is mainly on interventional therapy and patients with valvular heart disease (VHD) are often excluded from heart failure studies.

The primary focus in conservatively treated patients with a high burden of comorbidities is on the improvement of quality of life and progression-free survival. Regarding this, Sekuri et al. showed an improvement in exercise tolerance after six months of treatment with losartan. Furthermore, MR volume also showed a significant decrease [24]. In contrast, we did not implement quality-of-life measurements in our study. Nevertheless, the overall mortality was low in both groups and reduced numbers of heart failure-related hospital readmissions in the ACE-I/ARB group could be seen as an indicator of progression-free survival and improved quality of life.

### Study Limitations

Notably, there are several limitations in this study that must be acknowledged. First, the retrospective character clearly limits the amount and quality of the collected medical information. Further, although this is the largest study evaluating the effect of ACE-I/ARB in patients with moderate-to-severe MR, the overall number of the included patients remained low. However, our study authentically reproduces common patients in non-university medical centers in the sense of a real-world setting with a large number of frail patients and with no differentiation between secondary and primary MR. Our focus was not on candidacy for intervention. Many of our patients were not eligible for surgery or TEER, while in others the diagnosis of moderate-to-severe MR might be seen as a bystander as these patients were hospitalized for other reasons and the diagnosis of MR was not directly related to the admission diagnosis. In these cases, echocardiography was indicated for various reasons during the inpatient stay, which yielded the result of moderate- to high-grade MR. Ultimately, the significant differences in patients with dementia and high-grade aortic stenosis between the groups might lead to the assumption of medical undertreatment in these patients and selection bias.

We did not differentiate between ACE-I and ARB since ARB is recommended in the Guidelines if ACE-I are not tolerated [25]. We did not implement the occurrence of hypertension, but it has to be assumed that this was the main indication for ACE-I/ARB. The cost takeover of ARB by Austrian insurance companies is generally restricted to a contraindication of ACE-I, therefore we don’t see any bias in merging the groups. Since echocardiographic parameters were not reevaluated after one year, we cannot draw any conclusions about the possible hemodynamic effects leading to an outcome benefit in our treatment group.

## 5. Conclusions

The effect of ACE-I/ARB in patients with moderate-to-severe MR and reduced ejection fraction is sufficiently published. Nevertheless, there is no guideline-directed medical therapy for patients with preserved or mildly reduced EF. Our study showed improved outcomes in these patients when treated with ACE-I/ARB and ought to initiate a further investigation for the treatment of non-surgical candidates.

## Figures and Tables

**Table 1:** Baseline characteristics of the study population. Continuous variables are given as means ± SD. Categorial variables are given as frequencies and percentages.

		No ACE-I/ARB *n* = 92	ACE-I/ARB *n* = 84	*p*-Value
**Age—yrs**		82 (8)	81 (7)	0.38
**Male sex**		34% (31)	35% (29)	0.91
**Coronary artery disease**		21% (19)	24% (20)	0.61
**Diabetes mellitus**		18% (17)	20% (17)	0.77
**Atrial fibrillation**		70% (64)	62% (52)	0.28
**Extracardiac Arteriopathy**		37% (34)	26% (22)	0.13
**Chronic lung disease**		14% (13)	12% (10)	0.66
**CCS class 4 angina**		5% (5)	4% (3)	0.55
**NYHA**	I	59% (54)	50% (42)	0.56
	II	11% (10)	15% (13)	
	III	20% (18)	19% (16)	
	IV	11% (10)	15% (13)	
**Renal insufficiency**	Normal (>85 mL/min)	26% (24)	20% (17)	0.47
	Moderately impaired (50–85 mL/min)	55% (51)	55% (46)	
	Severely impaired (<50 mL/min)	18% (17)	25% (21)	
**Dementia**		14% (13)	5% (4)	0.034
**EuroScoreII (%)**		7 (8)	6 (7)	0.49
**Polypharmacy ≥ 5 prescriptions**		74% (68)	93% (78)	<0.001
**Oral anticoagulation**		64% (58)	62% (52)	0.80
**Beta blocker**		65% (60)	64% (54)	0.90
**Heart rate at admission**		82 (22)	79 (23)	0.40
**Heart rate at discharge**		73 (9)	71 (10)	0.13
**Frailty**		43% (40)	38% (32)	0.47
**Reason for admission**	Heart failure	40% (37)	39% (33)	0.69
	Gastroenterology	16% (15)	11% (9)	
	Other cardiac	18% (17)	17% (14)	
	Infection	12% (11)	14% (12)	
	Other	13% (12)	19% (16)	

Abbreviations: CCS Canadian Cardiovascular Society; GFR Glomerular Filtration Rate; CFS: clinical frailty scale.

**Table 2:** Echocardiographic parameters at baseline. Continuous variables are given as means ± SD. Categorial variables are given as percentages and frequencies.

		No ACE-I/ARB *n* = 92	ACE-I/ARB *n* = 84	*p*-Value
**Mitral regurgitation**	Moderate	72% (66)	68% (57)	0.57
	Severe	28% (26)	32% (27)	
**Vena contracta—mm**		6 (1)	6 (2)	0.062
**PISA**		8 (4)	8 (3)	0.74
**LVEDD—mm**		48 (8)	48 (9)	0.95
**Left Atrium—cm^2^**		29 (8)	29 (11)	0.88
**LVEF**	Mildly reduced	33% (30)	35% (29)	0.79
	Preserved	67% (62)	65% (55)	
**Aortic stenosis**	Absent	74% (68)	83% (70)	0.06
	Mild	5% (5)	8% (7)	
	Moderate	11% (10)	7% (6)	
	Severe	10% (9)	1% (1)	

Abbreviations: LVEDD Left Ventricular End-Diastolic Diameter; LVEF: left ventricular ejection fraction; PISA Proximal Isovelocity Surface Area.

**Table 3:** Univariate and multivariable Cox regression proportion hazard model.

	Univariate Model-1			Multivariable Model-2			Multivariable Model-3		
	HR	95%CI	*p*-Value	aHR	95%CI	*p*-Value	aHR	95%CI	*p*-Value
No ACE-I/ARB	Reference			Reference			Reference		
ACE-I/ARB	0.52	0.27–0.99	0.046	0.52	0.27–0.99	0.049	0.51	0.26–1.01	0.053

Abbreviations: HR hazard ratio; CI Confidence Interval.

## Data Availability

Not applicable.
